# Implications of the Immune Landscape in COPD and Lung Cancer: Smoking Versus Other Causes

**DOI:** 10.3389/fimmu.2022.846605

**Published:** 2022-03-21

**Authors:** Elisabeth Taucher, Iurii Mykoliuk, Joerg Lindenmann, Freyja-Maria Smolle-Juettner

**Affiliations:** ^1^ Division of Pulmonology, Department of Internal Medicine, Medical University Graz, Graz, Austria; ^2^ Division of Thoracic Surgery, Department of Surgery, Medical University Graz, Graz, Austria

**Keywords:** immune landscape, COPD, lung cancer, smoker, never-smoker

## Abstract

Cigarette smoking is reported in about one third of adults worldwide. A strong relationship between cigarette smoke exposure and chronic obstructive pulmonary disease (COPD) as well as lung cancer has been proven. However, about 15% of lung cancer cases, and between one fourth and one third of COPD cases, occur in never-smokers. The effects of cigarette smoke on the innate as well as the adaptive immune system have been widely investigated. It is assumed that certain immunologic features contribute to lung cancer and COPD development in the absence of smoking as the major risk factor. In this article, we review different immunological aspects of lung cancer and COPD with a special focus on non-smoking related risk factors.

## 1 Introduction

Tobacco smoking is reported in about one third of the adult population worldwide (1, 2). Smoke from tobacco is made up of various toxic and carcinogenic compounds, as for instance nicotine, nitrogen oxides and cadmium or carbon monoxide (3, 4). A relation of cigarette smoke exposure to cancers, respiratory-, cardiovascular-, infectious- and neurologic diseases has been proven (5–8). Immune function is considerably altered by smoking, which promotes the secretion of inflammatory mediators, comprising pro-, but also antiinflammatory cytokines (2, 9–12). Chronic inflammation and autoimmunity are two among many systemic effects of smoking (2, 3, 11, 13).

Lung cancer, as the number one cause of cancer-related deaths worldwide, is mainly a consequence of tobacco smoking (14–16). Still, there is a subgroup of lung cancer patients who have never smoked, and they differ from lung cancer patients who have smoked with respect to molecular markers and prognosis (15, 17, 18). About 10-15% of lung cancer cases occur in the never-smoking population (19–21). Known risk factors for lung cancer in never-smokers include second-hand smoking, indoor air pollution, occupational exposure to certain chemicals and a genetic predisposition (22, 23). However, certain immunological aspects may contribute to the susceptibility of never-smokers who develop lung cancer as well.

Chronic obstructive pulmonary disease (COPD) counts among the leading causes of morbidity and mortality worldwide, with the worldwide incidence still increasing (24, 25). Apart from tobacco exposure, which constitutes a major risk factor, other risk factors have also been outlined, including exposure to smoke from biomass fuel, occupational exposure to gases and dusts, a history of respiratory-tract infections in the early childhood or history of tuberculosis as well as air pollution (25, 26). Cigarette smoking is acknowledged as the single most important risk factor for COPD, yet, between one fourth and one third of COPD cases are attributed to lifetime never-smokers (27–30). Like in lung cancer, it remains unclear why certain non-smoking individuals develop COPD.

In this article, we review possible immunological mechanisms leading to lung cancer or COPD in the never-smoking population.

### 1.1 The Immune System in Elderly Patients

Lung cancer and COPD affect manly the elderly population (31, 32). In the elderly, the propensity for aberrant immune function increases significantly (33). The aging process goes along with changes of the innate, as well as the adaptive immune function (34, 35). Cancer and autoimmune disorders are encountered in elderly patients more frequently, which is partly due to age-related immunological changes, termed immunosenescence (36). Changes of the innate immunity include dendritic cell function, natural killer cells, neutrophil function and macrophages (37). Dendritic cells generally decrease in quantity during the aging process, like for example Langerhans cells in the skin or plasmocytes (38). Aged dendritic cells are characterized by dysfunction of their mitochondria, leading to a reduced ATP turnover and coupling efficiency as well as greater reactive oxygen species (ROS) production (39). Immunosenescene also leads to a dysfunctional maturation and function of natural killer cells (40). Changes in natural killer cell properties that are aging-related comprise a slower response to inflammatory conditions and a general increase of bacterial and fungal infections in the elderly population, which is due to the decline in natural killer cell quantity (41). During aging, spontaneous neutrophil apoptosis, mediated by the increased secretion of proinflammatory cytokines such as INK-1, occurs less frequently (42). Immunosenescence also impairs neutrophil migration to the lung tissue, as demonstrated in a mouse model (43). Insufficient neutrophil migration in elderly patients is most probably due to an increased constitutive PI3K activation, since lower rates of PI3K activation are normally observed in young individuals (44). Macrophages, like other cell types in aging persons, feature telomere shortening, which leads to a decreased GM-CSF-dependent proliferation of these cells, resulting from a decreased phosphorylation of STAT5. In aged mice, it was demonstrated that macrophages became increasingly susceptible to oxidative stress, explaining why DNA damage is caused by ROS to a much greater extent in old people, paving the way for malignant transformation (45). In elderly people, macrophages produce considerably lower amounts of cytokines such as TNF-α and IL-6, and show a reduced expression of the B7 receptor, resulting in a reduced activation of T-cells (46).

The adaptive immune system is also affected by immunosenescence, altering the function of B- and T-lymphocytes. Humoral immunity is impaired in aging patients, because of a relative increase of the memory B cells (IgG(+)IgD(-)CD27(-), double negative, DN) population, leading to a classical low-level chronic inflammatory microenvironment. Naïve/memory B-cell populations in older adults feature a different receptor expression, which has been discussed using the term “inflamm-aging” (47). Interestingly, naïve B-lymphocytes from young persons need very strong signals in order to be activated *in vitro*, whereas B-cells from elderly patients can be activated when only physiologically stimulated (48). The plasma cell count in the bloodstream of elderly patients was also observed to decline, as compared to young individuals (49). T-lymphocytes feature an altered functionality and distribution in old patients, which is why the susceptibility for infections and other diseases increases with age (50). A decline in naïve T-lymphocyte output is due to an age-related regression of the thymus (51). Another effect of thymus regression in the elderly is a reduced T-lymphocyte diversity (52).

Taken together, the above listed data shows distinct immunological differences in elderly, as compared to young patients. The increased prevalence of lung cancer and COPD in old subjects, smokers and non-smokers alike, is probably a synergistic event of cumulative noxa and immunosenescence.

## 2 The Effect of Smoking on the Immune Landscape

Smoking alters the immune response in different ways, paving the way for chronic bronchial inflammation and consecutively, lung cancer and COPD. In this chapter, we give an overview on the impacts of chronic cigarette smoke exposure on the immune system.

T-lymphocytes form one major subset of immune cells who mediate the adaptive immune response (2). Naïve T-lymphocytes may be activated and differentiated into effector T-cells, memory- and regulatory T-cells (53). The profound effect of tobacco smoking on T-cell function, including their secretion of proinflammatory mediators, has been shown by various studies (54, 55). One study was performed to analyze the occurrence of T-cells in bronchoalveolar lavage (BAL) fluid and in peripheral blood from non-smokers (n=40) and smokers (n=40) who had a normal lung function, compared with the result in COPD patients (n=38). CD8+ BAL cells were more abundant in smokers than in non-smokers (56). Meanwhile, CD4+ T-cells were found to be lower in the blood as well as the BAL fluid in the smoking group (56). Another study demonstrated a disruption of T-helper cell homeostasis in smokers who developed COPD when compared with healthy non-smokers (57). Second-hand smoking also affects the prevalence of T-cells, leading to an increase of CD3+ T-cells while active smoking increases CD8+ T-cells and lowers CD4+ T-cells (58). Moreover, in circulating T-cell subpopulations, the percentage of Th17 cells is increased in COPD patients, and the percentage of Th1 cells is also increased in COPD patients, and in current smokers without COPD as well (59). In a mouse model where mice were exposed to tobacco smoke for a minimum of six months, a significant elevation of Th1 and Th17 cells was observed (54). Taken together, murine as well as human studies indicate an increase of Th1 and Th17 cell subsets to result from chronic tobacco smoke exposure (2).

Cytotoxic T-lymphocytes (CD8+ cells) exert a main function in host immune defense, killing infected or otherwise impaired cells. In CD8 knockout mice, exposure to chronic cigarette smoke did not lead to an immune response, inflammation or emphysema (60). Furthermore, it was shown that IP-10 from CD8+ cells promoted elastase production from macrophages, consecutively resulting in elastin fragmentation and lung injury (60). Based on this result, it is assumed that CD8+ cells play a major role in the development of COPD. Another study demonstrated human CD8+ cells from COPD patients or from smokers without COPD to express more toll like receptor (TLR)4 and TLR9 proteins than controls. An enhancement in cytokine expression and general activation of circulating CD8+ T cells was also a direct effect of cigarette smoke exposure (61). A study on mice with pulmonary emphysema showed cigarette smoke to increase the percentage of IL-21+ Th17 and IL-21R+ CD8+ T-cells in peripheral blood, and to upregulate their expression of IL-17 and IL-21. Consecutively, perforin and granzyme B were upregulated in the CD8+ cells as well, showing a regulation of CD8+ T-cell function by Th17 cells in lung emphysema (62).

Also, regulatory T-cell function is disrupted by cigarette smoke exposure, as it was demonstrated in a study on BAL fluid from COPD patients or healthy smokers (63). An analysis of women smoking actively or passively during pregnancy showed a reduced regulatory T-cell count in the umbilical cord blood, leading to a higher amount of atopic dermatitis or food allergies in infancy (64).

According to epidemiologic studies, a higher prevalence of memory B-cells in the peripheral blood and memory IgG+ B-cells in the lung results from chronic smoking, when compared to non-smokers (65, 66). Cigarette smoke exposure downregulated B220+CD34− pre-B-cells and/or B220+CD34+ pro-B-cells in the bone marrow of mice (67, 68).

Memory T-cells (CD3+CD45RO+, CD4+CD45RO+) and class-switched memory B-cells are elevated as a consequence of chronic cigarette smoke exposure (65, 69–71). Interestingly, other data suggests the opposite effect of cigarette smoking on memory T-cells, namely a significant reduction of CD3+CD45RO+ and CD4+CD45RO+ memory T-cells in the blood of children exposed to second-hand smoke, accompanying with augmented percentages of CD3+ and CD4+CD45RA+ naive T-cells (58).

Not surprisingly, cigarette smoking has been linked to aberrations in the innate immune system as well (72–74). One study showed that exposure to cigarette smoke led to an elevation of IL-33 secretion from epithelial cells and changed the expression profile of IL-33 cognate receptor ST2 in various immune cells (75). In the same study, an enhanced proinflammatory response of macrophages and natural killer (NK) cells in case of inflammation, due to an augmented expression of ST2 was shown (75). Cigarette smoking caused inflammatory responses mediated by neutrophils and monocytes in a murine model, despite activated CD4+ T-cells being present in the murine lungs (76). This finding indicates that the innate immunity alone may cause acute inflammation as a response to smoke stimulation (76). It has been shown that TLRs are significantly involved in inflammatory response mechanisms caused by smoking (77). For instance, an analysis of patients suffering from periodontitis showed smoking to upregulate mRNA expression of TLR2 and TLR4 in the gingival tissue (78, 79). The results of studies investigating the effect of cigarette smoking on dendritic cells, however, are contradictory, because it has been found that cigarette smoke can suppress as well as promote dendritic cell development both in humans and mice (2, 80, 81). The differences in the immune response of smokers and non-smokers were illustrated by a study on the response of primary bronchial epithelial cells to pseudomonas aeruginosa lipopolysaccharide (82). Primary bronchial epithelial cells from smokers and non-smokers were compared *in vitro*. Bronchial epithelial cells from 16 patients with COPD, 10 healthy smokers and 9 non-smokers were cultured and exposed to cigarette smoke, prior to stimulation with pseudomonas aeruginosa lipopolysaccharide. It was found that more IL-8 and IL-6 was released from the COPD cultures than from cells of healthy smokers or non-smokers. Interestingly, pre-treatment with cigarette smoke reduced the release of IL-8 from the COPD patients’ bronchial epithelial cells, however, led to an increase of IL-8 release in the cells of smokers without airway obstruction and of non-smokers. After cigarette smoke treatment, TLR4 expression, mitogen activated protein kinase (MAPK) and NF-kB activation went down in COPD cultures, but not in the other two groups (82). It is therefore concluded that exposure to tobacco smoke decreases the inflammatory response to pseudomonas aeruginosa lipopolysaccharide in patients with COPD, but not in non-smokers or smokers without airflow limitation (82).

Mounting evidence suggests that the MAPK signaling pathway is strongly involved in COPD pathogenesis, contributing to a chronic inflammatory state by cell chemotaxis, remodeling of the bronchi and alveoli, insensitivity to corticosteroid treatment and airflow limitation (83, 84). The knowledge about dysfunctional MAPK signaling *in vivo* in COPD led to the investigation of MAPK inhibitors as possible COPD therapeutics, aiming to resolve in particular the aberrant neutrophil apoptosis observed in COPD (85, 86). Also for NF-kB, an involvement in COPD/chronic airway inflammation has been demonstrated *in vivo* (87). Therefore, NF-kB has increasingly become the focus of interest, outlining it as a potential therapeutic target in COPD treatment, and moreover, in the context of COVID-19 pneumonia (88).

The above listed data shows the profound impact cigarette smoking has on the immune function, affecting the innate as well as the adaptive immune response. Certain immunological properties, resulting from risk factors of pulmonary inflammation other than cigarette smoke, may specifically contribute to COPD and lung cancer in lifetime non-smokers.

## 3 Non-Smoking Related Risk Factors and Their Impact on the Immune Response

### 3.1 Air Pollution and Oxidative Stress

#### 3.1.1 The Impact of Air Pollution and Oxidative Stress on COPD Development

Air pollution is one of the main causes of COPD in lifetime non-smokers. Acute airway obstruction, exacerbations of asthma and COPD and a general increase in emergency department visits have been reported as a consequence of exposure to particulate air pollution (89, 90). Rudell et al. published a study where the adverse effect of diesel exhaust on the airways was investigated (90). 10 healthy and never-smoking individuals were exposed to diluted diesel exhaust without a ceramic particle trap for one hour, three times, with several weeks between exposures. 24 hours after the exposures, bronchoalveolar lavage was performed. The particle trap reduced the number of particles found in the BAL fluid. However, diesel exhaust caused a significant increase in neutrophil count in the lavage fluid. *In vitro*, diesel exhaust led to a reduction of phagocytosis by alveolar macrophages after diesel exhaust exposure with or without particle trap. In the absence of the particle trap, phagocytosis was reduced to a greater extent. Diesel exhaust also led to a migration of alveolar macrophages into the airspace, and a reduction in CD3+ CD25+ cells was observed (90). These immunologic events may contribute considerably to chronic airway inflammation in non-smokers upon chronic exposure to outdoor air pollution.

Oxidative stress is a main predisposing factor for the development of COPD (91). In smokers, antioxidant capacity is obviously reduced due to cigarette smoking, but also in non-smokers ROS from outdoor air pollution can lead to immunological changes that endorse COPD development. In COPD patients, alveolar macrophages are highly activated when compared to healthy subjects. These macrophages release increased amounts of ROS in the form of superoxide radical and hydrogen peroxide (92). Activated neutrophils from the peripheral blood from patients with COPD exert similar effects, releasing increased quantities of ROS as well, especially during COPD exacerbations. Markers of oxidative stress, like hydrogen peroxide, carbon monoxide and myeloperoxidase, as well as markers of oxidative tissue damage such as 8-isoprostane and carbonyl stress were shown to be upregulated in the exhaled breath from COPD patients (93–96). More than 50 cytokines and chemokines were demonstrated to be associated with COPD. Intracellular signaling pathways which are triggered by – or themselves drive – the release of inflammatory mediators, are susceptible to oxidative stress (91). Continuous inflammation in the lung tissue contributes considerably to COPD progression, and therefore a resolution of the inflammatory response is equally as important in COPD treatment as its induction. In this context, the clearance of apoptotic cells by phagocytosis plays a key role. However, in COPD patients, phagocytosis is impaired, which contributes to ongoing chronic lung inflammation (97, 98). Oxidative stress by ambient air pollution or other sources impacts the phagocytosis capacity, either intracellularly through changes in cytoskeletal organization (99), or extracellularly by the carbonylation of tissue proteins, leading to a competition for the same pattern recognition receptors (PRRs) expressed on alveolar macrophage surfaces that normally recognize and clear carbonyl-modified proteins and apoptotic cell detritus (100). Carbonylation caused by ROS was shown to modify and impair the function of PRRs as well, further impeding phagocytosis (101). Moreover, oxidative stress is responsible for the suppression of proinflammatory genes by corticosteroids in COPD patients (102). Carbonylation and nitration caused by air pollution leads to a reduced activity and expression of histone deacetylase 2 (HDAC2), a prime transcriptional corepressor of activated inflammatory genes and the antiinflammatory properties of corticosteroids (103, 104). According to these data, oxidative stress from air pollution can cause an increase in the expression of inflammatory genes, a failure to resolve the inflammatory response, insensitivity to corticosteroids and a diminished ability to induce endogenous antioxidant defense mechanisms. All these factors contribute to a more rapid aging of the lung (105), and may ultimately cause COPD in never-smoking subjects exposed to constant air pollution.

A study by Wooding et al. was conducted to show the immunologic mechanisms of COPD caused by outdoor air pollution in non-smokers (106). It has been shown previously that neutrophils are recruited to the lung as a consequence of diesel exhaust exposure. 18 individuals, amongst them never-smokers (n=7), ex-smokers (n=4) and patients with mild to moderate COPD (n=7), were included into this analysis. Following two hours exposure to diesel exhaust and two hours exposure of filtered air on separate occasions, neutrophil function in the blood was measured. The amount of circulating neutrophils was reduced after diesel exhaust exposure as compared to filtered air (106). The proportion of neutrophil extracellular traps in the lung was increased in all participants of this study. The authors confirmed these findings with *in vitro* experiments, showing that diesel exhaust particles led to an increase of neutrophil extracellular traps in isolated neutrophils. These data indicate the formation of neutrophil extracellular traps as a distinct mechanism contributing to airway obstruction and COPD formation in non-smokers.

#### 3.1.2 Air Pollution and Lung Cancer

Apart from COPD and chronic respiratory inflammation, a high risk of lung carcinogenesis has been attributed to the exposure to airborne particulate matter (PM) and ozone. PM are a group of different compounds, where the particle core is variable and there is a vast array of surface-related features, like heavy metals (107, 108). Ambient air pollution with PM under the size of 2.5 μm in diameter has been found to be a causative factor for lung tumors (108–110). Particles from air pollution can penetrate into the airways and persist there as particle deposits. PM from combustion sources cause the generation of ROS, leading to DNA damage. Most notably in this context are transition metals with redox capacity, persistent free radicals and redox-cycling of quinones, which may be activated to ROS, causing bulky adducts or strand breaks of the cellular DNA (111, 112). The effects of diesel exhaust particles have been investigated in numerous studies, showing that chronic exposure results in oxidative stress and radical-induced oxidative lesions. 8-Oxo-7,8-dihydroguanine (8-oxoGua) can be used as a biomarker for oxidative stress, measured by high-performance liquid chromatography (HPLC) and gas chromatography–mass spectrometry (GC-MS) in biomonitoring studies (108, 113–115).

Ozone has been reported as a pulmonary irritant, causing redox effects and leading to chronic oxidative stress in the lung tissue (116). Ozone exposure leads to an increased 8-hydroxy-2′-deoxyguanosine (8-OHdG) and heme oxygenase-1 (HO-1) expression in alveolar macrophages (117). According to epidemiological studies, individuals who are exposed to combined air pollutants, e.g. ozone in addition to second-hand smoke or PM, are at a considerably increased risk of pulmonary diseases due to chronic oxidative stress and inflammation (118). Oxidative stress is the key factor in PM-related airway disease, since ROS caused by inhalation of PM are involved in lipid peroxidation, DNA damage and oxidative damage of enzymes (119, 120). Antioxidants are crucial to counteract damage which is – to some degree – continuously done by ROS. Antioxidants allow for a maintenance of the redox balance, mediating biotic and antibiotic stress response and impact gene expression (108, 121). ROS created from airborne PM were demonstrated to activate MAPK family members as well as transcription factors, such as NF-κB and AP-1 (activator protein 1). By activation of these molecules, apoptosis, proliferation, differentiation and ultimately, malignant transformation are affected (122). Notably, ROS are not only generated as a direct effect of airborne PM, but also by target pulmonary epithelial cells or macrophages upon contact with PM (123). Resident macrophages from the lungs and alveolar spaces can release ROS after phagocytosis of inhaled PM, which leads to signal cascades contributing to inflammation and other pathobiological damage (124). Phagocyte-generated ROS comprise, amongst others, superoxide anion (O2−·), nitric oxide, free radical (NO·) and hydrogen peroxide (H2O2) (125). Macrophages are the prime cellular source of NO· in the lungs, while neutrophils form the second largest group. The enzyme inducible nitric oxide synthase (iNOS) serves as an immunological defense mechanism, generating NO· radicals from L-Arginine, NADPH and oxygen (126). ROS and RNS are essential for host defense, yet, they can cause considerable damage to nucleic acids, cellular lipids and proteins. A vast range of diseases is associated with an increase in ROS, like cardiovascular disease, neurological disorders, chronic inflammatory conditions, pulmonary fibrosis and cancer (127). Carcinogenesis is promoted by ROS because of DNA base modifications, rearrangement of DNA sequences, miscoding of DNA lesions, gene duplications and the activation of oncogenes (128). ROS also cause indirect oncogenic effects that lead to cancer, like the upregulation of hypoxia response genes, e.g. elevated levels of hypoxia-inducible transcription factor (HIF-1α) (129).

Oxidative stress results from constant exposure to ambient air pollution, and can be a causative factor for lung carcinogenesis in never-smokers. A study by Ito et al. was specifically designed to evaluate, why the prevalence of primary lung cancer has been increasing in never-smokers despite the decrease in smokers in the developed world (130). They investigated the impact of oxidative stress, possibly caused by outdoor air pollution, in never-smoking individuals with primary lung cancer. Never-smokers, smokers with more than 20 pack years and patients with benign lung diseases were included into the analysis. Serum oxidative stress and antioxidant capacity (AOC) were examined, and the oxidative damage on DNA in the lung tissue was examined. AOC was found to be significantly lower in never-smokers than in smokers. DNA damage was most often found in smokers, and also never-smokers with lung cancer were found to have significantly more DNA damage than subjects with benign lung tumors (130). The authors conclude that a low AOC in never-smokers with lung cancer might explain why some individuals are especially susceptible for lung cancer due to air pollution, even in the absence of smoking as a prime risk factor. The relation of long-term exposure to air pollution with lung cancer has been widely acknowledged. Yet, there is also a short-term association between air pollution and lung cancer mortality, which was examined in a Chinese study (131). Data on the daily prevalence of PM with a diameter <2.5 μm (PM2.5), and PM with a diameter <10 μm (PM10), sulfur dioxide (SO2), and ozone (O3), was assessed. These data were correlated to lung cancer mortality by time-series generalized linear models, correcting for meteorological factors as a possible confounder. According to this study, there is a positive association of the concentrations of PM2.5 and PM10, as well as SO2 with lung cancer mortality on a given day (131). Moreover, daily ozone exposure correlated with short-term lung cancer mortality as well. It was found that each 10 μg/m3 increase of any pollutant was linked to an excess risk of mortality, with a 7.16% increased risk of mortality at the highest concentrations. This study also highlights the important role of oxidative stress caused by air pollution in lung cancer, not only promoting carcinogenesis but also mortality from preexisting lung cancer. Another analysis from China was conducted to investigate the association between spatial air pollution and lung cancer incidence (132). SO2 had the greatest effect on lung cancer incidence in the north of China. In the south, all investigated pollutants (PM2.5, PM10, SO2, NO2, CO and O3) were significantly linked to an increased lung cancer incidence (132). The authors of this study also point out that smoking has a significant synergistic negative effect together with air pollution (132). Air pollution and the consecutive oxidative stress and associated immunological changes should therefore be seen as important contributing factors to lung cancer particularly in never-smokers, while increasing the lung cancer risk even further in the smoking population.

Oxidative stress from ambient air pollution or smoking seems to be the most widely investigated form in the context of lung cancer and COPD. Still, other sources of free radicals, like for instance, nutrition- or exercise-related ones, may alter the immune landscape and contribute to lung disease (133). Moderate exercise seems to be beneficial in the treatment of lung cancer and COPD (134), however, it must be kept in mind that strenuous activity as such leads to increments in ROS (135) and may actually be harmful. Inadequate or excessive nutrient intake also promotes or causes oxidative stress, disrupts oxidative homeostasis and activates molecular pathways that alter the immune landscape in a pro-carcinogenic fashion (136). Nutritional oxidative imbalance has been outlined as a risk factor for cancer, however, the exact immunological aspects in this context remain to be elucidated in further detail.

### 3.2 Occupational Exposure to Chemicals

#### 3.2.1 COPD as a Result of Exposure to Occupational Lung Irritants

Chronic pulmonary inflammation and COPD may also occur as a response to inhaled toxins, like mycotoxins and airborne particles of asbestos, silica and heavy metals (137). These toxins cause cell injury, which, as mentioned previously, poses a risk for lung carcinogenesis. In addition, cellular response to toxic particles leads to the initiation of immune-mediated repair processes. Consecutively, sustained inflammation and lung tissue remodeling leads to COPD. Especially in developing nations, burn biomass fuels, domestic fires and toxic particles from occupational exposure are a major risk factor for COPD (138). Lung inflammation as a response to toxins is a complex process, involving epithelial cells lining the airways and alveoli and immune cells from the bloodstream (137). Immune cells also secrete cytokines and chemokines, as well as growth factors, that endorse tissue restructuring and fibrosis (139, 140). During the acute inflammatory phase, neutrophils migrate to the lungs as first-line response, producing ROS, secreting serine proteases, matrix metalloproteinases and other proinflammatory enzymes. Theses secondary molecules lead to alveolar destruction which may result in COPD (141).

#### 3.2.2 Occupational Lung Carcinogens

In subjects with a history of silica and asbestos exposure, follow-up studies have suggested an increased risk of lung cancer later in life (142, 143). As early as the 1960s it was shown that workers from the textile industry exposed to high amounts of asbestos dust carried a 10-fold increased risk of lung cancer compared to the general population (144). In a European multi-center study, it was confirmed that exposure to silica dust leads to a two-fold increased lung cancer risk (145).

Generally, chronic exposure to silica and asbestos causes pneumoconiosis, also known as silicosis and asbestosis, ultimately leading to progressive lung fibrosis (146, 147). Silica and asbestos particles, after inhalation, are taken up by alveolar macrophages, resulting in an increase in the amount of collagenic fibroblasts and fibrous tissue remodeling surrounding the inhaled particles (148–150). The impact of silica dust leads to aberrations in antitumor immunity, contributing to the increased lung cancer risk in subjects with chronic silica dust exposure. The most critical change happens on the level of the macrophage, as shown for example by Rakhmilevich et al. (151, 152). In this study it was demonstrated that macrophages can be activated by CD40 ligation, consecutively exerting direct cytotoxicity against cancer cells. Anti-CD40 monoclonal antibody treatment could block tumor growth effectively, however, this effect was annihilated by silica exposure (151, 152). The reduction in macrophage count by silica could therefore promote lung carcinogenesis in non-smokers. Another effect leading to reduced antitumor immunity in non-smokers exposed to silica dust is the induction of macrophage and neutrophil infiltration into lung tissue, resulting in an enhanced secretion of cytokines, chemokines and ROS (150). In a murine model, injected silica led to a variety of immunologic changes, including the recruitment of splenic macrophages and neutrophils, reductions in B-lymphocyte levels as well as alterations in T-lymphocyte abundance (153). Silica also caused a five-fold increase in IL-12 release, and antigen presentation to T-cells *in vitro*, as well as the priming of antigen-specific T- and B-lymphocytes, was largely inhibited. Therefore, despite an abundance of IL-12, macrophage functions which are mandatory for antitumor immune response are blocked due to silica (154, 155). It has been acknowledged that silica dust exposure causes FasL overexpression in lung tissue (156, 157). Therefore, it is assumed that FasL leads to an increase of macrophage apoptosis in subjects who are exposed to silica dust, allowing for an unimpeded proliferation of cells transformed by silica (150). Fas/FasL affects macrophage function, rendering them unable to activate neither themselves, nor other cells that are critical to recognize and remove malignant cells. The interference with normal macrophage function is one key mechanism by which lung carcinogenesis is endorsed in non-smokers exposed to silica dust. According to the current literature, FasL overexpression due to silica leads to an increased release of TNF-α and other proinflammatory cytokines. However, despite newly-recruited immune cells due to cytokine release, no effective defense against already transformed cells is possible. This can be explained by an increased presence of transforming growth factor-β (TGF-β), resulting in a shift of FasL from an inflammatory function to a suppressive one, as shown by Chen et al. (158). In this study it was demonstrated that an increment in TGF-β blocked neutrophil activation by FasL and led to a reduced recognition of tumor cells by immune cells (158). Silica dust leads to an increased TGF-β expression, as shown by several studies (159, 160). It is thus assumed that the tumor-promoting effect of silica dust is mediated by TGF-β, mitigating the function of FasL towards a decreased release of cytokines or chemokines that are crucial for antitumor immunity, and causing a shift in FasL-associated activities from an inflammatory to a suppressive function, so that the removal of silica-induced transformed cells is largely impeded (150).

Chronic occupational exposure to asbestos and log-term inhalation of asbestos fibers has been linked to lung-, and specifically pleural carcinogenesis (161). The function of NK-cells and CD8+ cytotoxic T-lymphocytes is altered by asbestos fibers (162, 163), and since these cell types constitute the first-line anticarcinogenic defense mechanism, directly killing malignant cells (164, 165), antitumor immune response of asbestos-exposed individuals is impaired, rendering them especially at risk for lung and pleural carcinogenesis after a latency period of 30-40 years (162, 166). A study on the human NK-cell line YT-A1 showed a significantly reduced expression of two NK-cell specific activating receptors and of the serine protease granzyme A usually secreted by NK-cells to directly kill cancer cells, after continuous exposure to asbestos (150, 167). Asbestos also reduced the expression of the activating NK-cell receptor NKp46 in NK-cells stemming from mesothelioma patients (150, 167). A study on the effect of asbestos fibers on T-lymphocyte function, where a T-lymphocyte cell line was exposed to high asbestos dosages, showed a progressive time- and dose-dependent apoptosis of the T-cells in culture (150). Moreover, an activation of the mitochondrial apoptosis pathway was demonstrated, in conjunction with ROS production and proapoptotic signaling *via* p38 and c-Jun N-terminal kinase, as well as the activation of caspases-9 and -3 (150). In line with this observations, similar effects on alveolar epithelial cells as well as pleural mesothelial cells were seen upon exposure to asbestos *in vitro* (168–171). Maeda et al. also examined the long-term effect of asbestos exposure on T-cells *in vitro*. They found that after one year of chronic exposure to the asbestos agent chrysotile, the cells acquired resistance to asbestos-induced apoptosis. In addition, an increased expression and secretion of IL-10 and of the anti-apoptotic molecule Bcl-2 was observed (150). The T-lymphocyte cell line with acquired asbestos resistance also featured an enhanced TGF-β production along with a reduced production of IFN-γ, TNF-α, and IL-6.

Aside from silica and asbestos, several other occupational agents have been classified as group 1 carcinogenic agents for lung cancer by the International Agency for Research on Cancer (172, 173). **Table 1** summarizes these carcinogens as well as the respective pathomechanisms in relation to lung cancer (adapted from Algranti et al. and Siemiatyck et al.) (173, 174).

**Table 1:** Group 1 occupational carcinogens for lung cancer.

Occupational agents or circumstances of exposure	Main activities related to exposure	Consequence of exposure
Tars, pitch, soot, schist and bitumen	Dusts stemming from these compounds, such as from street paving, waterproofing of roofs, oil extraction and charcoal production	These agents comprise a mixture of potentially carcinogenic polycyclic aromatic hydrocarbons
Arsenic	Respiratory exposure during work with arsenic pesticides, smelting of copper ore or other ores with arsenic contamination (e.g. bronze)	
Asbestos	Manufacture of artifacts of asbestos cement, mining, working with asbestos compounds like the installation of tiles and water tanks or the manufacture and installation of brakes and brake pads	Asbestos not only causes lung cancer but also pleural mesothelioma
Beryllium	Production of beryllium, manufacture and use of high-hardness grinding wheels, beryllium salts	Beryllium is also the cause for the chronic lung condition berylliosis
Bis(chloromethyl) ether; chloromethyl methyl ether	Chemical synthesis in general, intermediate substrate in pesticide- and resin manufacture	
Cadmium	Inhalative exposure during cadmium mining, production of nickel-cadmium batteries and pigments for paints	The chronic respiratory exposure to cadmium dust also causes pulmonary emphysema
Hexavalent chromium	Exposure to stainless steel welding fumes, exposure to chromic acid mists during electroplating, zinc manufacture	
Occupational exposure to mists and vapors from acids containing sulfuric acid	Vapors of sulfuric acids stemming from battery charging, metal cleansing, manufacture of chemicals/petrochemicals	
Occupation of painter	House painting, vehicle painting	The occupation of painter is associated with a variety of risk factors including dusts and fumes in surface preparation, exposure to metals used in paint pigments, anti-rust agents, resins and asbestos
Manufacture and repair of footwear, occupation in the leather industry	A statistical association between these professions and cancer of lung, larynx and nasal cavity has been observed	The cause/pathophysiology remain unknown; it is speculated that dusts from leather and chemicals used during leather tanning act as carcinogens
Coke manufacture	The preparation of coal coke for steel production is associated with lung cancer	Coke manufacture leads to a large quantity of fumes rich in polycyclic aromatic hydrocarbons
Aluminum manufacture	The industrial aluminum production exposes workers to tar fumes	Polycyclic aromatic hydrocarbons generated in primary aluminum production
Iron and steel production	The industrial iron and steel production leads to tar fume exposure	Exposure to iron-production associated metals and polycyclic aromatic hydrocarbons that evolve during smelting is carcinogenic
Mustard gas	A carcinogenic gas used as chemical weapon; extremely irritating and toxic	
Coal gasification	The production of gas from charcoal leads to tar fume exposure	
Nickel	Nickel particles from the nickel refining process; compounds stemming from stainless steel welding process	
Radon	Gold mining, iron (hematite) and uranium mining	Radon is a radioactive gas that stems from the isotopic decay of uranium and radium
Free crystal silica	All types of exposure that can lead to chronic silicosis	The chronic inflammatory process during silicosis is associated with lung carcinogenesis
Passive smoking	Several occupations; for example, barkeeper, working in offices with smoking exposure, etc.	
Talc with asbestiform fibers	Industrial handling or mining of silicate, talc and soapstone geologically contaminated with asbestos	Talc dust (silicate), contaminated with asbestos, has the same effect as exposure to asbestos

### 3.3 Pulmonary Fibrosis as a Predisposing Factor for Lung Cancer

Mounting evidence suggests that fibrotic lung conditions also predispose individuals to lung carcinogenesis. This was first assumed based on autopsy studies, where coexisting interstitial lung disease and lung cancer have been found (175). Idiopathic pulmonary fibrosis is the most common form of fibrotic lung disease (176). Many patients with pulmonary fibrosis also have a history of smoking, rendering them especially susceptible for lung cancer. Still, non-smokers with fibrotic lung disease seem to have a higher lung cancer risk as well, as idiopathic pulmonary fibrosis increases lung cancer risk by 7-20%, regardless of other risk factors (177). Fibrosis of the lung originates from perpetuated and excessive connective tissue remodeling in response to recurring alveolar microinjuries. Excessive deposition of components of the extracellular matrix leads to an irreversible remodeling of lung tissue (178, 179). Fibroblasts, during excessive and aberrant wound healing, respond by hyperproliferation and change to a pro-fibrotic phenotype resistant to apoptosis (178, 179). Activated fibroblasts are highly responsive to growth factors and cytokines, e.g. TGF-β, connective tissue growth factor, platelet-derived growth factor and IL-6 (179, 180). This chronic low-level inflammatory process leads to an increased risk of lung carcinogenesis in patients with pulmonary fibrosis. T helper cells type 1 and 2 (Th1/Th2) play an important role in the inflammatory phase of pulmonary fibrosis (181). It was demonstrated in a mouse model that depletion of T-lymphocytes by means of an anti-CD3 monoclonal antibody impeded the fibrotic lung remodeling (182). The role of regulatory T-cells in fibrotic lung disease has not been completely clarified. A reduction of regulatory T-cells in the peripheral blood and BAL of pulmonary fibrosis patients was shown in on study, while other data showed an increase of regulatory T-cells (183). A pro-fibrotic impact of regulatory T-cells was shown in one study, increasing TGF-β1 release and collagen deposit (184). However, in late stages of lung fibrosis, regulatory T-cells acted anti-fibrotic (184). It may be that regulatory T-cell dysfunction contributes not only to pulmonary fibrosis but also to lung carcinogenesis. Particularly in the early stages of tumor development regulatory T-cells play a crucial role. A mouse model of mutant Kras-driven lung adenocarcinoma showed that Kras transgenic mice who lacked FoxP3+ regulatory T-cells were 75% less likely to develop lung tumors (185). Tobacco smoke led to an increase in FoxP3+ lymphocytes, according to the mouse study, which poses one among many factors contributing to smoking-related lung cancer. The depletion of regulatory T-cells led to tumor cell death at early stages of lung carcinogenesis according to another mouse study (186), resulting in elevated levels of granzyme A and B, perforin and IFN-γ in infiltrating CD8+ T-cells. Correspondingly, Xiong et al. demonstrated that regulatory T-cell depletion was a protective factor in radiation-induced lung fibrosis (187). Viewing the data on regulatory T-cells and lung fibrosis, it is evident that they can exert pro- as well as anti-fibrotic roles, dependent on the stage of pulmonary fibrosis and the mutual interaction with other T-cells. Th9 and Th22 T-cells, producing IL-9 and IL-22 were also involved in fibrotic remodeling of the lung, exerting pro- and anti-fibrotic properties similarly to regulatory T-cells (188–190). Macrophages, as a source of proinflammatory and pro-fibrotic cytokines, have been linked to lung fibrogenesis as well (191). The role of macrophages in lung carcinogenesis has been widely investigated, showing the important role macrophages play in this context. Macrophages make up the majority of tumor-infiltrating immune cells and are a key link from inflammation to lung cancer (192). Two hypotheses on the effect of macrophages and lung carcinogenesis have emerged: An extrinsic pathway driven by inflammatory conditions increasing the risk for lung cancer, as well as an intrinsic one driven by genetic changes (192). Tumors triggered by chronic inflammation at distinct tissue sites are characterized by infiltrating leukocytes, predominantly macrophages, cytokines, chemokines and growth factors. Tumor-infiltrating macrophages secrete ROS and nitrogen intermediates, inducing DNA damage. In response to tissue damage at sites of chronic inflammation, inflammatory cytokines are released to recruit cells that initiate tissues repair. However, the cytokines endorse tumorigenesis themselves by inhibiting key enzymes (193). Two types of tumor-associated macrophages are known today: canonically activated M1 and alternatively activated M2 macrophages. Both in lung cancer and in lung fibrosis, these two subtypes contribute considerably to disease progression, exerting beneficial as well as harmful functions (194, 195). M2 macrophages have been known to accumulate in fibrotic lungs and have been linked to pro-fibrotic activities (194). Conversely, anti-fibrotic roles have been reported for macrophages as well, mediated by diverse mechanisms such as scavenging of proinflammatory cellular debris, digestion of extracellular matrix components and by secretion of mediators inducing myofibroblast apoptosis (196). The effects of macrophages both in fibrotic lung disease and in lung cancer may explain why lung fibrosis is linked to lung cancer, and why changes in the lung microenvironment associated with lung fibrosis render the patients more susceptible for lung cancer as well.

Neutrophils play a role in chronic inflammation in general, and their role in lung carcinogenesis as well as in lung fibrosis has been commonly acknowledged (197, 198). Levels of IL-8/CXCL8, which is a key chemotactic factor for neutrophil function, are increased in patients with pulmonary fibrosis (199), and the number of neutrophils in the BAL fluid correlated well with G-CSF levels, an important neutrophil growth factor (200). Airway neutrophils are generally activated at a higher level in idiopathic pulmonary fibrosis, compared with healthy individuals, which is demonstrated by increased levels of their main proteolytic product, neutrophil elastase (201). Neutrophil elastase is a protease of neutrophil extracellular traps which are formed as a response to chronic lung inflammation. One mouse study showed that neutrophil elastase proteolytically remodeled laminin in lung tissue, leading to the proliferation of dormant lung cancer cells *via* the activation of integrin α3β1 signaling (202). Tissue remodeling processes in lung fibrosis, mediated by neutrophils, could therefore facilitate concomitant lung carcinogenesis. Summing up all the above-mentioned data, a great variety of immunological changes can be linked to fibrotic lung disease. Many of these changes are generally associated with chronic inflammation or tissue remodeling, paving the way for malignant transformation of cells. The correlation of lung fibrosis with an increased risk of lung cancer – particularly in never-smokers – can partly be explained by these distinct immunological changes.

Mycotoxins and fungal spores are especially prevalent in damp buildings or at farms, in the malt and wood industry, i.e. all occupations that involve handling moldy materials (203). Different fungi are sources of mycotoxins as secondary metabolites, including trichothecenes synthesized by the genera Fusarium, Myrothecium, Trichoderma, Trichothecium, Cephalosporium, Verticimonosporium, and Stachybotrys (204). Trichothecenes lead to a suppression of lymphocyte-related immune response and stimulate the production of IL-1β by macrophages (205). Additionally, these toxins inhibit protein synthesis by targeting the ribosome, impairment of the mitochondria and activation of MAPKs (206). Trichothecenes also upregulate gene expression related to the inflammatory response, which leads to further ribosome damage through inflammatory cytokines (207, 208). Deoxynivalenol is a trichothecene which is found in contaminated cereal grains. It may exert cytotoxic effects and inflammation synergistically with particulate matter, inducing the inflammatory response (209). Animal studies have demonstrated that upon exposure, mycotoxins not only damage the lung but are distributed to the liver, kidney and spleen (210). Consecutively, alveolar macrophages and neutrophils are recruited, promoting pulmonary hemorrhage, cytokine secretion and ultimately, damage to various organs (211). Notable, even after ingestion of mycotoxins, the lung tissue is injured in response (212).

Ricin is a ribosome-inactivating protein found in beans of the castor plant Ricinus communis. Ricin aerosol does not occur naturally but may be used as a biological weapon because of its high toxicity. Ricin is used as a compound of immunotoxins to target cancer cells (213). Ricin intoxication leads to a strong inflammatory cascade, i.e. the migration of neutrophils to the airways, causing apoptosis and necrosis of lung epithelial cells (214). Conversely to cigarette smoke, ricin also causes apoptosis of alveolar macrophages (214). While severe ricin poisoning leads to interstitial pneumonia, alveolar edema, respiratory failure and death within days, a sublethal ricin dosage leads to lung fibrosis and pulmonary hemorrhage (215). Like cigarette smoke and mycotoxins, ricin increases the expression of proinflammatory genes in airway epithelial cells and pulmonary macrophages (137). Both ricin and mycotoxins are factors predisposing exposed individuals to fibrotic lung disease. The above-listed inflammatory mechanisms contribute not only to fibrosis, but to DNA damage of lung epithelial cells as well, which may lead to lung cancer in the long term. Lastly, also non-biological chemicals can lead to lung inflammation when inhaled. These include volatile organic compounds from household items, office supplies and craft materials, e.g. formaldehyde, benzene and perchloroethylene, and chronic exposure results in damage of the lung epithelium, fibrotic remodeling and malignant transformation (216).

### 3.4 Second-Hand Smoke, COPD and Lung Cancer

Second-hand smoke counts among the risk factors for COPD in non- and never-smokers (217). Similarly, second-hand smoke, also termed “environmental tobacco smoke”, has been classified as a main pulmonary carcinogen (218). Second-hand smoke is a composition of mainstream- and sidestream smoke, wich are mostly similar in quality and composition (219), yet, sidestream smoke arises at lower burning temperature and the quantities of its chemical compounds in vapor and particular phase (i.e. the quantity of semi-volatile or non-volatile agents) differ from the quantities in mainstream smoke (220). Certain carcinogens, such as aromatic amines, are more abundant in sidestream smoke (220, 221). Second-hand smoke, once released into the air, has the potential to aggregate and mix with other types of air pollutants (222). Smokers are generally exposed to a much larger quantity of smoke-related carcinogens (221). Interestingly, sidestream smoke was associated with a higher potency to induce mouse skin tumors, which gave rise to the assumption that second-hand smoke in non-smoking subjects might even be more carcinogenic than mainstream smoke inhaled by smokers (223).

Since data on second-hand smoke, COPD and lung cancer is gathered epidemiologically, the evidence linking second-hand smoke to these conditions remains weak (224), and a direct biological link is difficult to outline. In a mouse study, it was shown that alveolar macrophage recruitment was increased after exposure to second-hand smoke in a dose-dependent fashion (217). Moreover, an increased expression of alveolar macrophage markers and an increase of the COPD-associated pro-inflammatory markers CCL2 and TNFalpha was also observed in this study (217), showing a direct causative effect of second-hand smoke in COPD.

In lung carcinogenesis, second-hand smoke is an established risk factor (22, 225), however, attempts to epidemiologically confirm the associated risk have only brought forth disputable evidence (226). In COPD and lung cancer, the uncertainties in risk assessment of second-hand smoke exposure have arisen, because exposure assessment is difficult to standardize, given temporal variabilities in source, formulation and concentration of effluent second-hand smoke (227). However, for lung cancer, the risk does significantly increase due to second-hand smoke with many years of latency (228). Novel studies have focused on outlining second-hand smoke biomarkers with the aim to measure exposure over a relevant time period which augments the risk of lung carcinogenesis (221, 229). Human epidemiologic studies on second-hand smoke and lung cancer have been observational (226), correlating the incidence of lung cancer in long-term second-hand smoke exposed individuals, such as family members of smokers, to the level of second-hand smoke exposure (230). Rodent studies on the carcinogenic potential have been performed, allowing for a better standardization, however, animals are usually treated with considerably higher doses of smoke than humans would be exposed to in real life (231). Moreover, animals with smoke exposure often respond with appetite suppression and the consecutive inadequate caloric intake may further contribute to tumor initiation (231). Thus, it is difficult to exactly quantify the carcinogenic potential of passive smoking.

Very few data exists on the immunologic changes induced by second-hand smoke. Presumably this is due to the assumption that the changes are very similar to those caused by active smoking. One mouse study investigated *in utero* and early-life passive smoke exposure, and the resulting immunologic changes (232). Changes in plasma cytokine concentrations and in immune cell populations, i.e. decreased percentages of B-cells and increased percentages of myeloid cells were observed in the bone marrow of smoke-exposed versus control mice. In the spleen of passive smoke exposed mice, the abundance of B-cells decreased and that of T-cells increased, whereas myeloid cells were significantly lower in the peripheral blood in smoke exposed mice (232). The authors of this study conclude that the immune changes resulting from second-hand smoke *in utero* and in early life are particularly threatening, contributing to carcinogenesis by lowering host defense mechanisms. In a review article about the immunologic changes of second-hand smoke in humans, it becomes evident that the literature is yet too limited to draw conclusions on how exactly the immunologic changes induced by second-hand smoke differ from that of active smoking (233). Environmental smoke exposure may induce hypersensitivity, leading to asthma and may furthermore suppress immunoregulatory mechanisms. Some studies even failed to detect any immunological changes in passive smoke-exposed individuals (233). Thus, more research is clearly warranted on this topic.

### 3.5 Pulmonary Infections and the Lung Microbiome Are Related to Lung Carcinogenesis

There is evidence for an increased risk of lung cancer due to a history of pulmonary infections caused by Mycobacterium tuberculosis (234). This evidence is supported by large cohort studies, like for instance a Chinese analysis amongst individuals with a history of tuberculosis infection. A positive correlation with lung adenocarcinoma as well as squamous cell carcinoma has been shown (235). According to previous data, the risk of lung cancer is increased by about 50% after a prior diagnosis of tuberculosis (236). The more recently the infection was diagnosed, the greater the risk of lung cancer became (236). Moreover, it is discussed whether a chronic pulmonary colonization with Chlamydia pneumoniae led to an increment in the risk of lung cancer as well (237). As shown by serologic testing, a chronic Chlamydia pneumoniae infection resulted in impaired apoptosis of the infected cells, because IL-10, a prime immunosuppressive cytokine, was activated, resulting in reduced antitumor immune response (238). Moreover, an upregulation of IL-8, mediated by Chlamydia pneumoniae, has been reported to increase the risk of malignancy, endorsing angiogenesis and cell proliferation, as shown in a mouse model (239).

The lung microbiome in general seems to impact the likelihood of lung cancer, in smokers and non-smokers alike (240). Apart from chronic inflammation and bacterial and viral lung infections, even periodontal disease and not only pathogenic but also opportunistic microorganisms can drive lung carcinogenesis in the long term, as shown for Haemophilus influenzae, Enterobacter spp., E. coli, Pneumococcus (241), Legionella (242) and Moraxella genera (137, 243). These pathogens can increase the likelihood of lung cancer, with an association even with certain histological subtypes (240). Klebsiella, Rhodoferax, Acidovorax, Comamonas and Polarmonas genera, for example, are frequently found in the lung microbiome of small-cell lung cancer (SCLC) patients, but not in patients with adenocarcinoma (244). It is proposed, that in some cases not the infection itself increases the risk for subsequent lung cancer, but the significant disruption of the lung microbial landscape. Alpha diversity of the lung microbiome, namely the number of different microbes in one habitat, is usually low in lung cancer patients. Interestingly, the beta diversity, meaning the diversity of microbes between habitats is not different when comparing healthy lungs and those from lung cancer patients (245). In recent years, the importance of the microbiome as an indicator of malignant transformation was recognized increasingly. A study on the lung microbiome, comparing samples from patients with benign and malignant tumors *via* next generation sequencing, showed the genera Veillonella and Megasphaera to be potential lung cancer biomarkers (243). Moreover, a correlation between Acidovorax genus and SCLC has been shown (244), and Pseudomonas is associated with lung adenocarcinoma (240, 246). Within the last decades, the molecular mechanisms by which microbes endorse malignant transformation, have been investigated in greater detail. The microbial carcinogenic effects comprise bacterial toxins, inflammatory stimuli from immune cells and the direct effects of microbes on epithelial cells. A study on mice with a dysfunctional lung microbiome and impaired function of γδT17 T-cells due to long-term antibiotic treatment, showed an increased risk for Lewis lung carcinoma. The total bacterial count of the lung microbiome in these mice decreased dramatically, showing that the commensal microbiome is indeed crucial for anti-tumor immune cell functions (247). Also bacterial toxins change the immune landscape in a way that may contribute to tumorigenesis. For instance, cytolethal distending toxin (CDT), cytotoxic necrotizing factor 1, and Bacteroides fragilis toxin can damage the DNA repair system, allowing for malignant transformation (248–250). In a murine model of A427 non-small cell lung cancer (NSCLC) it was demonstrated that the toxin of Cyanobacteria decreases CD36 protein levels and increases the concentration of PARP1, an enzyme profoundly linked to tumor development. In the mouse model, these results were proven with bacteria-positive lung cancer (251). Moreover, heat-inactivated E. coli bacteria were found to enhance migration and metastatic dissemination of NSCLC cells *in vivo* (252). Apart from these direct effects of microorganisms on lung cancer development, it was also shown that a shift in the lung microbiome causes ROS rates to increase. Higher amount of ROS lead to an increase in DNA damage in the long term and predispose to tumor development. Interestingly, TP53 mutated lung tumors are known to be associated with the colonization with Acidovorax genus in the tissue microenvironment (244).

A disruption of the lung microbiome, triggered by lung infections or possibly chronic exposure to carcinogenic dusts or second-hand smoke, may therefore be an important factor predisposing non- and never-smokers for lung cancer.

## 4 Conclusion

The prime risk factor for lung cancer and COPD is tobacco smoking. However, in non- and never-smokers, these diseases also occur because of other noxa not related to tobacco smoke. When comparing the effects of smoking on the immune landscape with the damage on lung tissue caused by ambient air pollutants, oxidative stress in general, occupational toxins and DNA damage as a side effect of lung fibrosis or pulmonary infections, the complex changes of the immune landscape are partly overlapping. Recently it was discovered that the lung microbiome also has a profound effect on the immune response, carcinogenesis and tissue remodeling, which remains to be investigated in greater detail. It can be said that smoking has a synergistic adverse effect when co-occurring with the above-mentioned other risk factors.

**Table 2** contains the most important bullet points of this manuscript, summarizing non-smoking related risk factors for lung cancer and COPD (**Table 2**).

**Table 2:** Take-home messages of this review article.

Non-smoking related risk factor	Lung cancer	COPD
Air pollution	Airborne PM and ozone lead to chronic respiratory inflammation and create ROS, paving the way for lung cancer PM persist in the airways as particle deposits Particles from diesel exhaust lead to radical-induced oxidative lesions Day-to-day mortality from lung cancer is associated with airborne PM readings	An increase in emergency department visits of COPD and asthma patients is observed as a consequence of increased particulate matter readings Diesel exhaust is particularly related to COPD development, reducing phagocytosis of alveolar macrophages
Oxidative stress	Ozone, *via* redox effects, causes chronic oxidative stress in the lungs The exposure to a combination of air pollutants (ozone and PM) leads to a synergistic increase of lung cancer risk Pulmonary antioxidant capacity, as a crucial tumor-suppressive event, is significantly impaired by ROS	ROS from ambient air pollution cause immunological changes leading to lung tissue damage Activated macrophages and neutrophils from the bloodsteam of COPD patients release ROS as well, further promoting COPD development Oxidative stress from air pollution increases the expression of proinflammatory genes, damaging the alveoli
Asbestos	Asbestos exposure is associated with an increased lung cancer, and especially, pleural mesothelioma risk Alveolar macrophages harbor inhaled asbestos fibers, and an increased amount of collagenic fibroblasts and tissue remodeling surrounding these fibers is observed Immune cell functions, and the associated anti-tumor defense mechanisms, are impaired due to chronic asbestos exposure	Chronic asbestos exposure leads to lung tissue remodeling, ultimately resulting in COPD Asbestos and other occupational lung irritants cause an increased secretion of cytokines and chemokines from immune cells, as well as growth factors responsible for lung tissue damage
Silica	The anti-tumor activity of alveolar macrophages is largely impeded by silica exposure The loss of normal pulmonary macrophage function is one of the key events leading to lung cancer upon silica dust exposure In mouse models, silica also led to a reduction in B- and T-lymphocytes, and a reduction in antigen-presentation and priming of antigen-specific T- and B-lymphocytes	
Pulmonary fibrosis	Fibrotic lung conditions predispose patients to lung cancer Fibrosis leads to a chronic low-level inflammatory state Excessive connective tissue remodeling and alveolar microinjuries render the lung tissue more susceptible for malignant transformation Smoking, in addition to lung fibrosis, synergistically augments lung cancer risk	
Pulmonary infectons	Pulmonary infections caused my Mycobacterium tuberculosis increase the risk for lung cancer later in life Colonization with Chlamydia pneumoniae may also increase the risk for malignant transformation	
Lung microbiome	Alterations of the lung microbiome by recurrent bacterial and viral infections (e.g. in immunosuppressed patients) may facilitate malignant transformation in the lung tissue Periodontal disease and opportunistic microorganisms have been found to alter the lung microbiome, posing a possible risk factor for lung cancer as well Haemophilus influenzae, Enterobacter spp., Pneumococcus, Legionella and Moraxella genera count among the microbes that have been linked to lung carcinogenesis	

## Author Contributions

ET, concept and structure of this article and main part of manuscript writing. IM, assistance in writing and proofreading. JL, in-depth proofreading, assistance in writing and continuous input during the writing process. F-MS-J, in-depth proofreading, final remarks and revision. All authors contributed to the article and approved the submitted version.

## Conflict of Interest

The authors declare that the research was conducted in the absence of any commercial or financial relationships that could be construed as a potential conflict of interest.

## Publisher’s Note

All claims expressed in this article are solely those of the authors and do not necessarily represent those of their affiliated organizations, or those of the publisher, the editors and the reviewers. Any product that may be evaluated in this article, or claim that may be made by its manufacturer, is not guaranteed or endorsed by the publisher.
